# Phase 1 study of napabucasin, a cancer stemness inhibitor, in patients with advanced solid tumors

**DOI:** 10.1007/s00280-020-04059-3

**Published:** 2020-03-31

**Authors:** Akihito Kawazoe, Yasutoshi Kuboki, Hideaki Bando, Shota Fukuoka, Takashi Kojima, Yoichi Naito, Shuichi Iino, Yasuhide Yodo, Toshihiko Doi, Kohei Shitara, Takayuki Yoshino

**Affiliations:** 1 Department of Gastroenterology and Gastrointestinal Oncology Division, National Cancer Center Hospital East, Kashiwa, Japan; 2 Department of Experimental Therapeutics, National Cancer Center Hospital East, Kashiwa, Japan; 3 Sumitomo Dainippon Pharma, Osaka, Japan

**Keywords:** Advanced solid tumors, Napabucasin, Cancer stemness inhibitor, STAT3

## Abstract

**Purpose:**

Napabucasin is a cancer stemness inhibitor that targets a number of oncogenic pathways, including signal transducer and activator of transcription 3 (STAT3). Phase 1/2 studies suggest tolerability and anti-tumor activity in various types of cancer; a Phase 3 study of napabucasin plus standard therapy in colorectal cancer is ongoing. This is a Phase 1 dose-escalation study in patients with advanced solid tumors, and the first study of napabucasin in Japanese patients.

**Methods:**

Patients received napabucasin 480, 960, or 1440 mg daily in 28-day cycles until disease progression or intolerable toxicity. Primary objectives were to determine dose-limiting toxicities (DLTs), maximum tolerated dose (MTD), and the pharmacokinetic (PK) profile of napabucasin. Blood samples were taken for PK analysis on Days 1, 2, 8, and 15 of Cycle 1, and Days 29 and 30 of Cycle 2. Secondary objectives were to assess napabucasin antitumor activity, and the relationship between biomarkers and antitumor activity. JapicCTI-No: JapicCTI-132152.

**Results:**

Enrolled were 14 patients (480 mg [*n* = 3], 960 mg [*n* = 4], 1440 mg [*n* = 7]). One patient experienced a DLT (Grade 3, anorexia). MTD was 1440 mg/day. Most common drug-related adverse events were diarrhea (*n* = 9), nausea (*n* = 4), vomiting (*n* = 3), and anorexia (*n* = 3). Napabucasin showed a similar PK profile to previous studies and no abnormal accumulation was observed. Following treatment, two patients had stable disease; the remaining 12 had progressive disease.

**Conclusion:**

Napabucasin was well-tolerated at doses up to 1440 mg/day in Japanese patients with advanced solid tumors; the PK profile was comparable to that reported previously.

## Introduction

Cancer stem cells (CSCs) are a subset of cancer cells that have stem cell-like properties, in that they can drive cell proliferation and differentiation [1–3]. The CSC hypothesis suggests that CSCs promote tumor development, recurrence, metastasis, and therapy resistance [4]. CSCs could, therefore, be a target for cancer drug development.

Signal transducer and activator of transcription 3 (STAT3) is thought to play a key role in regulating CSCs in several types of cancer [5] and is involved in a number of cell signaling pathways implicated in cancer cell survival, proliferation, metastasis, and angiogenesis [6]. Furthermore, STAT3 has been shown to be constitutively active in several types of cancer, suggesting that it could be a viable therapeutic target [2, 7, 8].

Napabucasin is a small molecule that has been shown in a recent preclinical study to be bioactivated by NAD(P)H: quinone oxidoreductase-1, and to a lesser extent by cytochrome P450 oxidoreductase, which results in redox cycling and the generation of reactive oxygen species (ROS) [9]. The subsequent increase in ROS levels results in DNA damage and is hypothesized to affect multiple oncogenic cellular pathways, including inhibition of the STAT3 signaling pathway, which has been implicated in cancer stem cell viability [2]. Napabucasin has the potential to treat certain types of cancer by inhibiting the self-renewal and survival of cancer stem cells [2], as well as inducing apoptosis in both cancer stem cells and heterogeneous cancer cells [10]. Since cancer therapies often fail to treat metastatic disease, it is hoped that targeting CSCs with napabucasin may reduce cancer relapse [2]. Zhang et al. (2016) reported that napabucasin inhibited prostate cancer cell survival, motility, and tumorigenesis in vitro [11]. Furthermore, Li et al. (2015) found that, in a mouse model of cancer relapse, napabucasin selectively targets CSCs, leaving normal hematopoietic stem cells unaffected [2].

Based on the promising results observed in vitro and in animal xenograft models [2, 11], several napabucasin clinical studies have been initiated in patients with advanced or metastatic cancers. A dose-escalation study in adult patients (14 cohorts; *N* = 41) with advanced cancer showed that napabucasin was well tolerated at doses up to 2000 mg/day; the maximum tolerated dose (MTD) was not reached (no dose-limiting toxicities [DLTs]) and adverse events (AEs) were generally mild, the most frequent being grade 1–2 gastrointestinal toxicities [12]. The recommended dosing regimen for napabucasin was determined as 500 mg BID at the time of a Phase I extension study in patients with advanced solid tumors [13]. A further clinical study of napabucasin in combination with paclitaxel demonstrated tolerability and signs of antitumor activity in patients with gastric or gastroesophageal junction adenocarcinoma [14]. An international Phase 3 study of napabucasin monotherapy in patients with advanced colorectal cancer [15] and an international Phase 3 study of napabucasin in combination with paclitaxel in patients with advanced gastric and gastroesophageal junction adenocarcinoma [16] were planned and conducted. However, following the results of an intermediate analysis, both trials were suspended due to futility. Two further international Phase 3 studies were undertaken [17, 18] based on the promising results of Phase 1b/2 clinical studies with napabucasin in combination with standard treatment in patients with pancreatic and colorectal cancer [19, 20]. Both these Phase 1b/2 studies showed combination therapy with napabucasin 240 mg BID to be well tolerated, with Grade 1–2 diarrhea, nausea, vomiting, abdominal cramps and fatigue being among the most frequently reported AEs; there were no DLTs or notable pharmacokinetic (PK) interactions [19, 20]. While the Phase 3 colorectal cancer study is ongoing, the Phase 3 pancreatic cancer trial was suspended due to futility, based on the results of an intermediate analysis, despite positive early findings. The present Phase 1 dose-escalation study is the first study of napabucasin in Japanese patients with advanced solid tumors.

## Materials and methods

### Study design

This Phase 1, open-label, dose-escalation study (3 + 3 design) was carried out at the National Cancer Center Hospital East in Japan. Patients received napabucasin 240 mg twice daily (BID) (Cohort 1), 480 mg BID (Cohort 2), or 720 mg BID (Cohort 3). Day 1 was defined as the day the first dose of napabucasin was administered. All patients were hospitalized until PK assessments were completed (Day 30), though patients were permitted to go/stay out during this period at the discretion of the investigators. MTD was defined as the highest dose level at which ≤ 1 of six evaluable patients had a DLT. Dose escalation would continue if no DLTs were observed, and if one of the first three patients experienced a DLT within the first cycle an additional three patients would be enrolled. Dose escalation would be halted if ≥ 2 of the first three patients (or ≥ 2 of the six patients, if additional patients were enrolled) experienced a DLT. If ≤ 1 of the patients in Cohort 3 (highest dose) experienced an observable DLT, then three additional patients were enrolled and would receive the highest dose level of napabucasin, and DLTs would be evaluated in the additional patients. Study treatment was given in 28-day cycles until disease progression or intolerable toxicity due to AEs.

The primary objectives of the study were to determine DLTs, MTD, and the PK profile of napabucasin in patients with advanced solid tumors. Secondary objectives were to assess the antitumor activity of napabucasin and to examine the relationship between biomarkers and the antitumor activity of napabucasin in patients with advanced solid tumors.

The study was conducted in accordance with the Declaration of Helsinki and Good Clinical Practice guidelines. An ethics committee or institutional review board approved the final protocol at the study site. All patients provided written informed consent. This study was registered with JapicCTI (JapicCTI-No.: JapicCTI-132152).

### Patients

Male and female patients aged ≥ 20 years with confirmed malignancies were included if they met the following inclusion criteria: primary lesion evaluable according to RECIST 1.1; Eastern Cooperative Oncology Group Performance Status of 0 or 1 at registration and baseline assessment; major organ function, within 3 weeks prior to the first dose of napabucasin, satisfying the following criteria: hemoglobin ≥ 9.0 g/dL; neutrophils ≥ 1500/µL; platelets ≥ 100,000/µL; serum creatinine ≤ 1.5 × upper limit of normal (ULN); total bilirubin ≤ 1.5 mg/dL; AST and ALT ≤ 3.0 × ULN or ≤ 5.0 × ULN in presence of liver cancer or liver metastases; and life expectancy ≥ 3 months.

Patients were excluded from the trial if they: received chemotherapy or radiotherapy (except for pain control at a limited region) up to 14 days before the first dose of napabucasin (including the same day of the week, 2 weeks before); received hormonal therapy, immunotherapy, thermotherapy, operative therapy or other therapies with anti-tumor activity (including bisphosphonates and denosumab for the treatment of cancer-related bone lesions) for the underlying disease within 4 weeks of the first dose of napabucasin (including the same day of the week, 4 weeks before the first dose of napabucasin); experienced any brain metastases (symptomatic or requiring treatment); had Crohn’s disease, ulcerative colitis, extensive gastric or massive small intestine resection; had received other investigational products within 4 weeks of the first dose of napabucasin (including the same day 4 weeks before the first dose of napabucasin). Post-nausea/vomiting prophylactic anti-emetics were permitted.

### Pharmacokinetic assessments

PK variables included maximum and minimum concentration (*C*_max_ and *C*_min_), area under the curve during 24 h (AUC_0–24_), area under the curve from time 0 to infinity (AUC_0–∞_), time of maximum plasma concentration (*t*_max_), half-life (*t*_1/2_) and apparent total clearance (CL/F). Blood samples for PK were obtained on Days 1, 2, 8, and 15 of Cycle 1 and Days 29 and 30 of Cycle 2. On Day 1 of Cycle 1 and Day 29 of Cycle 2, blood samples were collected before the morning dose and at 2, 4, 6, 8, 10, 12, and 24 h after the morning dose of napabucasin; for samples collected 24 h following the morning dose of napabucasin on Day 1 and Day 29, collection occurred before the morning dose was administered on Day 2 and Day 30, respectively. On Days 8 and 15 of Cycle 1, blood samples were taken 168 and 336 h after the morning dose on Day 1. Urine samples were taken on Days 1 and 2 of Cycle 1 and Days 29 and 30 of Cycle 2. Samples were collected immediately after the morning dose on Days 1 and 29 through to immediately before the morning dose the next day. Plasma napabucasin concentration was measured by Frontage Laboratories and CMIC Pharma Science Co., Ltd and urine napabucasin concentration was measured by CMIC Pharma Science Co., Ltd.

### Safety assessments

Safety endpoints included AEs, drug-related AEs, vital signs, body weight, laboratory values, and 12-lead ECG. All details of AEs were recorded, including the severity of the event. The severity of AEs was classified according to CTCAE v4.0-JCOG. Napabucasin dose could be reduced or interrupted due to AEs if considered necessary by the investigators, and later reinstated if the AE recovered to Grade 2 or lower. It could also be permanently discontinued in patients who met the discontinuation criteria, which included AEs that rendered trial continuation difficult, aggravation of underlying disease, and withdrawal of consent. Body weight was measured at baseline and Day 29 of Cycle 2, and every 28 days in subsequent cycles. Vital signs were measured at baseline, Days 1, 2, 8, 15, and 22 of Cycle 1, Days 29, 36, 43, and 50 of Cycle 2, and every 28 days in subsequent cycles. 12-lead ECG was measured at baseline and Day 57 in Cycle 3, and every 28 days in subsequent cycles. Laboratory values were recorded at baseline, Days 8, 15, and 22 in Cycle 1, Days 29, 36, 43, and 50 in Cycle 2, and every 28 days in subsequent cycles.

### Assessment of antitumor activity

Assessments of antitumor activity were objective tumor response, median progression-free survival (PFS) and median overall survival (OS). Tumor response was evaluated according to RECIST 1.1 [21]. Median PFS was defined as the time from the day of the first dose of napabucasin to the date of progressive disease (PD), if neither was observed, the patient was to be censored at the last assessment of tumor response. Median OS was defined as the time from the first dose of napabucasin to the date of death from any cause. Patients who were still alive at the final observation were to be censored at the date they were last known to be alive. STAT3 single nucleotide polymorphism (SNP) genotyping was carried out using DNA extracted from whole blood samples to assess tumor response, median PFS, and median OS. Immunohistochemistry (IHC), mutation analysis (Oncomine Comprehensive Assay), and microsatellite instability analyses were performed in tumor tissue samples obtained from patients who provided additional consent to assess the relationship between specific biomarkers and the antitumor activity of napabucasin. IHC staining of phosphorylated STAT3 (pSTAT3) was carried out on archived tumor samples which were collected pre-treatment, and de novo lesions during and post-treatment were also collected, according to the protocol of a recent Phase 3 study [15]. Positive pSTAT3 staining was defined as cancer cell nuclear staining ≥ 5% plus a stroma staining score of at least 2. The biomarker testing facility was responsible for IHC staining and storage of tumor samples. If consent to a genetic test was withdrawn, samples and associated analyses were discarded.

As an exploratory endpoint, the relationship between napabucasin and post-treatment antitumor activity was evaluated in patients after the completion of study treatment. Response was evaluated based on tumor imaging scans, PFS, and OS.

### Statistical analyses

The safety and antitumor activity (intention-to-treat [ITT]) analysis populations included all patients that received at least one dose of napabucasin. The DLT analysis population included all patients that received at least one dose of napabucasin with at least 80% compliance ([number of capsules actually ingested/number of capsules that should have been ingested per dose × 55] × 100) during the DLT evaluation period, and those patients who experienced DLT during the evaluation period regardless of compliance. The PK population included all patients that received at least one dose of napabucasin and had plasma napabucasin concentration data for at least one-time point after the first dose of napabucasin.

## Results

### Patients (disposition and baseline characteristics)

A total of 14 patients were enrolled, 3 in Cohort 1, 4 in Cohort 2 and 7 in Cohort 3. Baseline characteristics are shown in Table 1. In total, 8 patients were male and 6 were female, age ranged between 45 and 76 years. Primary cancer sites were either the colon, rectum or adrenal gland, and all patients had metastatic disease. The majority of patients (13/14, 92.9%) had undergone prior surgery.

**Table 1:** Patient baseline characteristics

Characteristic	All patients (*n* = 14)
Median age, years (range)	61.5 (45–76)
Sex, *n* (%)	
Male	8 (57.1)
Female	6 (42.9)
Primary tumor type, *n* (%)	
Colon	8 (57.1)
Rectum	5 (35.7)
Adrenal gland	1 (7.1)
ECOG PS, *n* (%)	
0	13 (92.9)
1	1 (7.1)
Metastasis, *n* (%)	
Yes	14 (100.0)
No	0 (0)
Location of metastatic cancer	
Liver	9 (64.3%)
Other	12 (85.7%)
Prior surgery, *n* (%)	
Yes	13 (92.9)
No	1 (7.1)
Prior radiotherapy, *n* (%)	
Yes	1 (7.1)
No	13 (92.9)
Number of prior treatment regimens, *n* (%)	
2	3 (21.4)
≥ 3	11 (78.6)

*ECOG PS* Eastern Cooperative Oncology Group Performance Status

### Safety

Drug-related AEs are summarized in Table 2. Overall, 12 of 14 patients were included in the DLT population (two permanently discontinued the trial prior to DLT evaluation, one [Cohort 2] due to PD and one [Cohort 3] due to the withdrawal of consent). One patient out of 12 in the DLT evaluation population experienced a DLT, and napabucasin was permanently discontinued (Grade 3 anorexia, Cohort 3). The remaining 11 patients permanently discontinued the trial between Cycles 2 and 4 due to PD. MTD was determined to be 1440 mg/day. AEs were reported in all 14 patients in the safety analysis population. AEs led to dose interruptions in five patients (due to a combination of nausea, anorexia, vomiting, dehydration, fatigue, diarrhea, gastroenteritis, and abdominal distension). No patients died due to AEs and there were no dose reductions due to AEs. In total, 11 patients experienced drug-related AEs, the most common being gastrointestinal disorders; all incidences of diarrhea were drug-related. The initial onset of diarrhea and vomiting was most frequently observed 1–7 days after the first dose, while the initial onset of nausea was most frequently observed 1–14 days after the first dose. The reasons for discontinuation from the study for all 14 patients were as follows: PD in 12 patients (including one patient who discontinued prior to DLT evaluation, Cohort 2), AE in one patient (Grade 3 anorexia, Cohort 3) and withdrawal of consent for one patient (Cohort 3).

**Table 2:** Details of drug-related adverse events

Number of patients (%)	Cohort 1 (480 mg/day, *n* = 3)	Cohort 2 (960 mg/day, *n* = 4)	Cohort 3 1440 mg/day, *n* = 7)	Total
				*n* = 14
Any drug-related adverse event				
Grade 1	1 (33.3)	4 (100)	4 (57.1)	9 (64.3)
Grade 2			1 (14.3)	1 (7.1)
Grade 3			1 (14.3)	1 (7.1)
Gastrointestinal disorders				
Diarrhea				
Grade 1	1 (33.3)	3 (75.0)	4 (57.1)	8 (57.1)
Grade 3			1 (14.3)	1 (7.1)
Nausea (Grade 1)	0 (0.0)	0 (0.0)	4 (57.1)	4 (28.6)
Vomiting (Grade 1)	0 (0.0)	1 (25.0)	2 (28.6)	3 (21.4)
Abdominal pain lower (Grade 1)	0 (0.0)	1 (25.0)	0 (0.0)	1 (7.1)
Infections and infestations				
Urinary tract infection (Grade 1)	0 (0.0)	1 (25.0)	0 (0.0)	1 (7.1)
Metabolism and nutrition disorders				
Anorexia				
Grade 1	1 (33.3)	0 (0.0)	0 (0.0)	1 (7.1)
Grade 2	0 (0.0)	0 (0.0)	1 (14.3)	1 (7.1)
Grade 3	0 (0.0)	0 (0.0)	1 (14.3)	1 (7.1)
Skin and subcutaneous tissue disorders				
Rash (Grade 1)	0 (0.0)	1 (25.0)	0 (0.0)	1 (7.1)

### Pharmacokinetics

Plasma PK of napabucasin are shown in Table 3. On Day 1, maximum plasma concentration of napabucasin was reached 4–6 h after administration of napabucasin 240 mg BID (Cohort 1) and 2–6 h after administration of napabucasin 480 mg BID (Cohort 2) or 720 mg BID (Cohort 3). Variability of plasma napabucasin concentrations between patients was observed, but there was no substantial difference observed over the time-course, despite an increased trough after the dose on Day 28 compared to Day 1 of Cycle 1. The minimum and maximum values of *C*_max_ and AUC_0–12_ overlapped between cohorts, suggesting no clear difference between doses. Similarly, no clear differences were observed between cohorts in urinary PK, suggesting that napabucasin dose does not affect these parameters.

**Table 3:** Pharmacokinetic parameters of napabucasin in blood plasma

Cycle	Cohort (dose, mg/day)	Number of patients, n	*C*_max_, ng/mL, range	AUC_0–12,_ h*ng/mL, range	AUC_0–24,_ h*ng/mL, range	AUC_0–∞,_ h*ng/mL, range	*t*_1/2_, h, range	*t*_max_, h range	CL/F, L/h, range
1	1 (480)	3	194–468	1373–2991	2018–3802	3653–4059	5–25	4–6	59–66
	2 (960)	4	199–719	984–4780	1196–6361	1407–7364	7–8	2–6	65–341
	3 (1440)	7	641–2070	3441–10,998	3922–14,540	3873–14,829	3–12	2–6	49–186
2	1 (480)	3	472–652	2194–4944	2513–6184	2570–6369	4–5	4–10	49–109^a^
	2 (960)	3	391–477	2556–2756	2750–2881	2754–2883	2–3	2–10	174–188^a^
	3 (1440)	5	596–1930	3647–15,413	4482–20,199	4741–123,120	5–192	2–4	47–197^a^

^a^Apparent total clearance at steady state

AUC_0–12_, area under the curve 0–12 h; AUC_0–24_, area under the curve, 0–24 h; AUC_0–∞_, area under the curve, 0- infinity; CL/F, apparent total clearance; *C*_max_, maximum plasma concentration; *t*_1/2,_ elimination half-life; *t*_max_, time after administration when the maximum plasma concentration was reached

### Antitumor activity

The best overall tumor response was stable disease (SD), which was observed in two patients with colorectal cancer; one patient in Cohort 1 had SD for 56 days, and one patient in Cohort 3 had SD for 82 days. PD was observed in the remaining 12 patients. Median PFS was 1.87 months in Cohort 1, 1.76 months in Cohort 2, and 1.45 months in Cohort 3. Median OS was 8.28 months in Cohort 1, 5.03 months in Cohort 2, and 5.68 months in Cohort 3. In the ITT population, median PFS was 1.71 months and median OS was 5.83 months.

STAT3 SNP analysis suggested that there was no difference in antitumor activity between G/G, C/C, and C/G genotypes. Two patients with colorectal cancer consented to an additional biomarker assessment and an investigation of pSTAT3 status. pSTAT3 status was negative for the first patient at pre-treatment. The second patient received trifluridine and tipiracil (TAS-102) post-napabucasin treatment and demonstrated a partial response (PR) 8 weeks after the completion of study treatment, which was maintained for 17 weeks. pSTAT3 status was investigated at three-time points in tumor samples obtained from this patient (Table 4). pSTAT3 status was positive pre- and post-napabucasin treatment, and negative post-TAS-102 treatment.

**Table 4:** pSTAT3 IHC staining of a patient who achieved a PR post-treatment with napabucasin

	Percent nuclear tumor staining				% Nuclear tumor positive	H-score	Microenvironment	
	0	1 +	2 +	3 +			% positive	intensity
Baseline	94	5	1	0	6	7	20	2
Post-napabucasin	90	5	5	0	10	15	50	2
Post-TAS-102	98	2	0	0	2	2	30	2

Post-napabucasin: Day 26 from the first dose of napabucasin. Treatment with napabucasin was ongoing at the time of sample collection. Post-TAS-102: Day 70 from TAS-102 first administration

pSTAT3-positivity was defined as cancer cell nuclear staining of ≥ 5% plus a stroma staining score of at least 2

*IHC* immunohistochemistry, *PR* partial response, *TAS-102* trifluridine and tipiracil

## Discussion

In this Phase 1, open-label dose-escalation study of napabucasin in Japanese patients with advanced solid tumors, one patient included in the DLT analysis population experienced a DLT (Grade 3 anorexia); MTD was determined to be 1440 mg/day. Drug-related AEs were predominantly gastrointestinal in nature with onset most frequently occurring in the first 2 weeks after first dose. These findings are generally in line with previously reported safety profiles of napabucasin [19, 22] and the findings from an earlier Phase I study in non-Japanese patients, which recommended at the time that the dosing regimen for napabucasin should be 500 mg BID [13]. Furthermore, another dose-escalation study reported similar AEs but did not observe any DLT at 2000 mg/day, therefore no MTD was determined [12].

PK analyses revealed no substantial differences in plasma napabucasin concentration over the time-course of the study, eliminating the possibility of abnormal accumulation. For both blood and urinary PK parameters, minimum and maximum values of *C*_max_ and AUC_0–12_ overlapped between cohorts, suggesting that they are not affected by napabucasin dose. Previous napabucasin studies also revealed similar PK profiles with no significant pharmacokinetic findings [12, 20]. It must be noted that these studies were conducted in the US and Canada, suggesting that the PK profile of napabucasin is comparable in Japanese and non-Japanese patients.

The best overall tumor response was SD, which was observed in two patients, while PD was observed in the remaining 12 patients. Langleben et al. observed SD as the best response in their Phase 1 dose-escalation study in a higher proportion of patients (65% of patients) [12]. This study demonstrated similar results to an international Phase 3 study of napabucasin in patients with colorectal cancer, 12% of patients achieved SD, compared with 14% in this study [15].

As CSCs are resistant to conventional therapies [4], we hypothesize that concomitant treatment with napabucasin (targeting both tumor and CSCs) may have higher antitumor activity than napabucasin or conventional therapies alone. One study of napabucasin in combination with paclitaxel showed tolerability and signs of anti-cancer activity in breast cancer [23], and several Phase 1b/2 studies have reported complete or partial tumor responses when administering napabucasin in combination with monoclonal antibodies or chemotherapy [19, 22, 24]. Additionally, one study of napabucasin plus FOLFIRI (folinic acid [leucovorin], fluorouracil, and irinotecan) showed the potential of napabucasin to sensitize cancer cells to FOLFIRI in colorectal cancer [20]. A Phase 3 study is currently recruiting patients across North America, Europe, Australia, and Asia with metastatic colorectal cancer [17] to assess the efficacy of napabucasin in combination with chemotherapy regimens.

In the present study, the above-mentioned patient with colorectal cancer treated with TAS-102 post-napabucasin treatment showed a PR at 8 weeks which was maintained at 17 weeks in the post-treatment period. In this patient, pSTAT3 status was positive at baseline and during napabucasin treatment, but negative after treatment. No new genetic mutations were observed. Based on these data, it could be hypothesized that napabucasin may increase patients’ susceptibility to chemotherapy via downregulation of pSTAT3 expression; however, further research is needed.

In conclusion, in the first study of napabucasin in Japanese patients, napabucasin was tolerated at 1440 mg/day in Japanese patients with advanced solid tumors. Studies are ongoing in various types of cancer to elucidate the potential of napabucasin as a cancer stemness inhibitor.
